# p70S6K promotes IL-6-induced epithelial-mesenchymal transition and metastasis of head and neck squamous cell carcinoma

**DOI:** 10.18632/oncotarget.9282

**Published:** 2016-05-11

**Authors:** Dandan Wu, Jie Cheng, Geng Sun, Shengjie Wu, Min Li, Zhongyuan Gao, Sulan Zhai, Ping Li, Dongming Su, Xuerong Wang

**Affiliations:** ^1^ Department of Pharmacology, Nanjing Medical University, Nanjing, Jiangsu Province 210029, China; ^2^ Department of Basic Medicine, Kangda College of Nanjing Medical University, Lianyungang, Jiangsu Province 222000, China; ^3^ Department of Oral and Maxillofacial Surgery, Affiliated Hospital of Stomatology, Nanjing Medical University, Nanjing, Jiangsu Province 210029, China; ^4^ Center for Clinical Pathology and Laboratory, Affiliated Hospital of Yifu, Nanjing Medical University, Nanjing, Jiangsu Province 211166, China; ^5^ Department of Pathology, Nanjing Medical University, Nanjing, Jiangsu Province 210029, China; ^6^ Key Laboratory of Human Functional Genomics of Jiangsu Province, Nanjing Medical University, Nanjing, Jiangsu Province 210029, China

**Keywords:** p70S6K, IL-6, epithelial-mesenchymal transition, HNSCC, metastasis

## Abstract

Head and neck squamous cell carcinoma (HNSCC) is the fifth most common cancer worldwide and a common cause of cancer-related death, with a 5-year survival rate of less than 60%. IL-6 has been suggested to play an important role in cancer metastasis, but its mechanism in HNSCC has not been fully clarified. p70S6K has been reported to induce epithelial-mesenchymal transition (EMT) of ovarian cancer, but its role in HNSCC remains unknown. In this study, we found that p70S6K and IL-6 were upregulated in high-metastatic HNSCC cell lines that underwent EMT when compared to paired low-metastatic cell lines. Overexpression of p70S6K promoted EMT and migration of HNSCC cells, while downregulation of p70S6K attenuated IL-6-induced EMT and cell migration. Furthermore, IL-6-induced p70S6K activation was attenuated by inhibitors of the PI3K/Akt/mTOR, MAPK/ERK, and JAK/STAT3 signaling pathways, suggesting that it located downstream of these pathways. These findings suggest that p70S6K promotes IL-6-induced EMT and metastasis of HNSCC. Targeting p70S6K for HNSCC therapy may benefit patients through the inhibition of tumor growth, as well as metastasis.

## INTRODUCTION

Head and neck squamous cell carcinoma (HNSCC) is the fifth most common cancer worldwide and a common cause of cancer-related death [1]. It arises from epithelia of the upper aerodigestive tract, including the oral cavity, oropharynx, larynx, or hypopharynx [2]. Currently, the treatments for HNSCC, including surgery, radiotherapy, cytotoxic chemotherapy, and/or molecular-targeted cancer therapy have improved significantly, but the outcome is very limited [1]. The 5-year survival rate of patients with early stage localized HNSCC was more than 80%, while the survival rate decreased to 40% for patients with neck lymph node metastasis, and it decreased to less than 20% for patients with distant metastasis [3]. Due to most patients being diagnosed in the late stage with metastasis, the overall survival rate of HNSCC patients is very poor [4]. Therefore, identifying and clarifying those molecules that play important roles in the metastasis of HNSCC are urgently needed for improving the early diagnosis and the selection of effective therapeutic strategies.

Epithelial-mesenchymal transition (EMT) is the first and most critical step in tumor metastasis [5]. Tumor cells undergoing EMT lose their epithelial markers, such as E-cadherin, and express mesenchymal markers, such as vimentin and/or N-cadherin. The migratory and invasive ability of these cells are increased to facilitate their detachment from the initial sites [6]. EMT has been proved to promote cell proliferation, survival, differentiation, cancer stem cells' “stem feature,” and drug resistance [7]. EMT plays important roles in cancer malignancy, metastasis, and reoccurrence. To date, the precise molecular events that promote EMT in head and neck cancer are poorly understood [8].

Interleukin 6 (IL-6), a glycoprotein that consists of 184 amino acids, is a pleiotropic cytokine that plays important roles in immune response and inflammation [9]. IL-6 can also be synthesized, expressed, and secreted by cancer cells, except inflammatory cells. In the tumor microenvironment, IL-6 activates its receptor (IL-6R) on the cancer cell membrane and promotes the development of cancer in the manner of paracrine and autocrine [10, 11]. IL-6's overexpression, dysregulation, and elevated serum levels have been proven to be associated with many types of cancers, such as multiple myeloma, lung cancer, ovarian cancer, breast cancer, and head and neck cancer [4, 12–14]. Furthermore, IL-6 promotes drug resistance, and elevated IL-6 serum levels predict poor drug responses [10, 15, 16]. In HNSCC, activation of STAT3 by IL-6 and EGFR has been proven to be a key molecular event [1, 4]. Other downstream signals of IL-6 include PI3K/Akt/mTOR and MAPK/ERK. Yadav et al. reported that IL-6 promoted EMT and metastasis of HNSCC via the JAK-STAT3-SNAIL signaling pathway [8]. However, the molecular mechanism of IL-6-induced EMT and metastasis in HNSCC has not been fully elucidated.

The 70-kDa ribosomal S6 kinase (p70S6K) is a serine/threonine kinase and belongs to the AGC-kinase family, which includes Akt, the protein kinase C (PKC), and the 90-kDa ribosomal S6 kinase (p90RSK) [17, 18]. As a well-known downstream target of mTORC1 (mammalian target of rapamycin complex 1), it promotes cell growth through increasing global protein synthesis by increasing ribosomal production to facilitate mRNA translation [19]. p70S6K is a downstream signal of the PI3K/Akt/mTOR and MAPK/ERK signaling pathways, and it mediates crosstalk between these two pathways in multiple regulatory levels. The PI3K/Akt/mTOR and EGFR/MAPK/AP-1 signaling pathways are proven to have significant effects on malignant phenotypes of HNSCCs [4, 20, 21]. Pon et al. reported that p70S6K promotes EMT through snail induction in ovarian cancer cells [22]. However, the role and mechanism of p70S6K in EMT and in the metastasis of HNSCCs remains unknown.

In this study, we first examined the expression of p70S6K and IL-6 in paired high- and low-metastatic HNSCC cell lines. Then we investigated the role of p70S6K in IL-6-induced EMT and in the migration of HNSCC cells. Finally, we explored the molecular mechanism of IL-6-induced activation of p70S6K.

## RESULTS

### IL-6 was upregulated in high-metastatic HNSCC cells compared to low-metastatic cells, and it promoted EMT and cell migration

**Figure 1:**
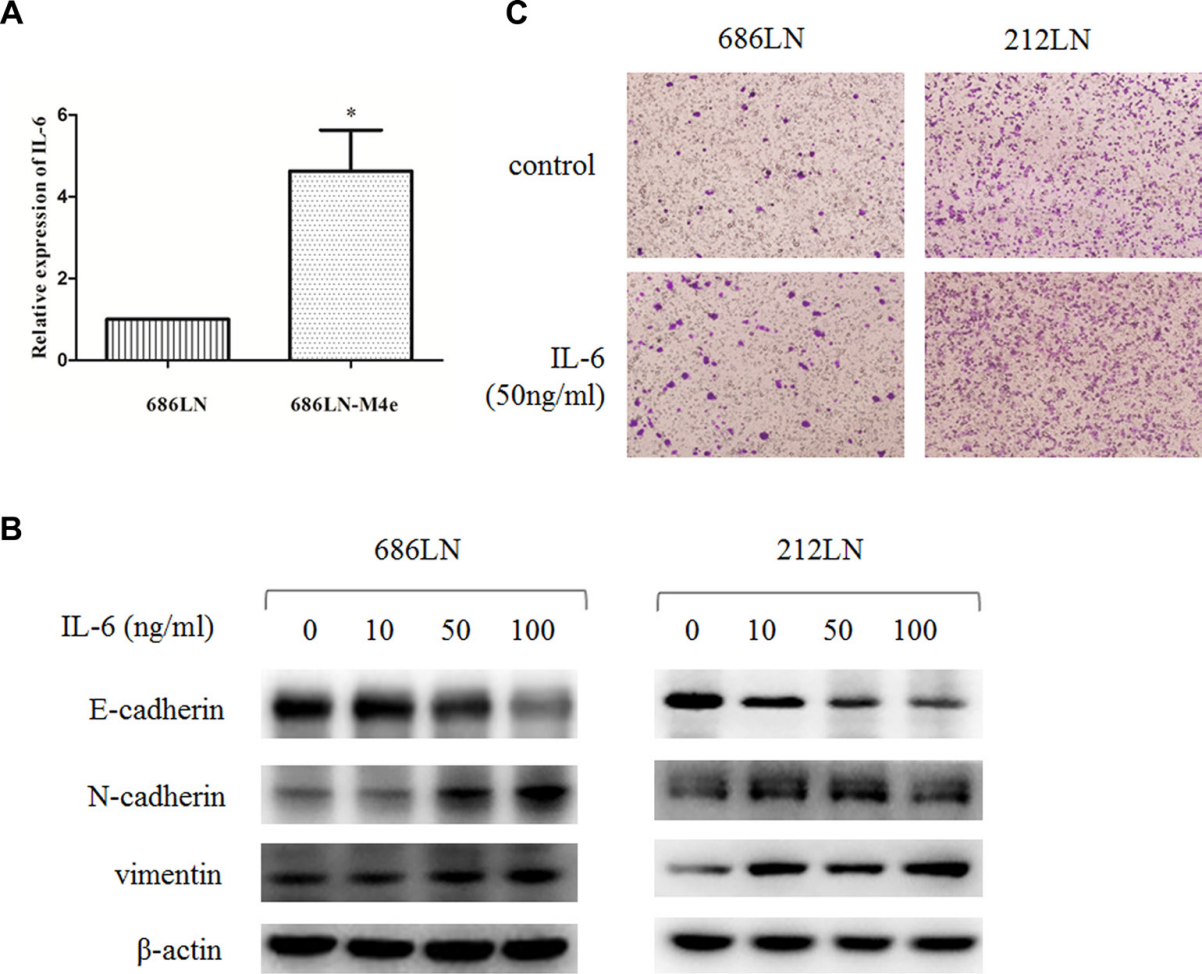
IL-6 is upregulated in 686LN-M4e cells compared to 686LN cells, and IL-6 induces EMT and migration (**A**) total mRNAs of 686LN and 686LN-M4e cells were prepared and subjected to qRT-PCR assay. Columns, mean of three replicate determinations; bars, SD. *p < 0.05. (**B**) 686LN and 212LN cells were serum starved overnight, then stimulated with different concentrations of IL-6, as indicated, for 48 hours. The whole-cell protein lysates were prepared and subjected to western blot analysis. (**C**) 686LN or 212LN cells were seeded into the chambers in the 24-well plates with serum-free medium. Then, the medium in the outside of the chamber was replaced with condition medium containing 50 ng/ml IL-6 or its vehicle for another 24 hours and subjected to transwell assay. Cells on the bottom side of the chamber were recorded under a microscope. Magnification: 100×.

IL-6 has been reported to be a key cytokine that promotes the malignancy of HNSCCs. Yadav et al. reported that IL-6 promoted the metastasis of HNSCCs by inducing EMT [8]. Therefore, we first detected the expression of IL-6 in our paired high- and low-metastatic HNSCC cell line models. The qRT-PCR assay showed that the mRNA levels of IL-6 were upregulated in high-metastatic 686LN-M4e cells compared to low-metastatic 686LN cells (Figure 1A). Furthermore, a western blot assay showed that IL-6 decreased E-cadherin, while it increased N-cadherin and vimentin expression in two HNSCC cell lines (686LN and 212LN) in a dose-dependent manner, suggesting that IL-6 induced EMT (Figure 1B). Finally, transwell assay showed that IL-6 promoted the migration of 686LN and 212LN cells (Figure 1C). These results suggest that elevated IL-6 levels may induce EMT and promote the metastasis of HNSCCs in our cell line model.

### p70S6K was upregulated in high-metastatic HNSCC cells compared to low-metastatic cells, and IL-6 activated the p70S6K signaling pathway

**Figure 2:**
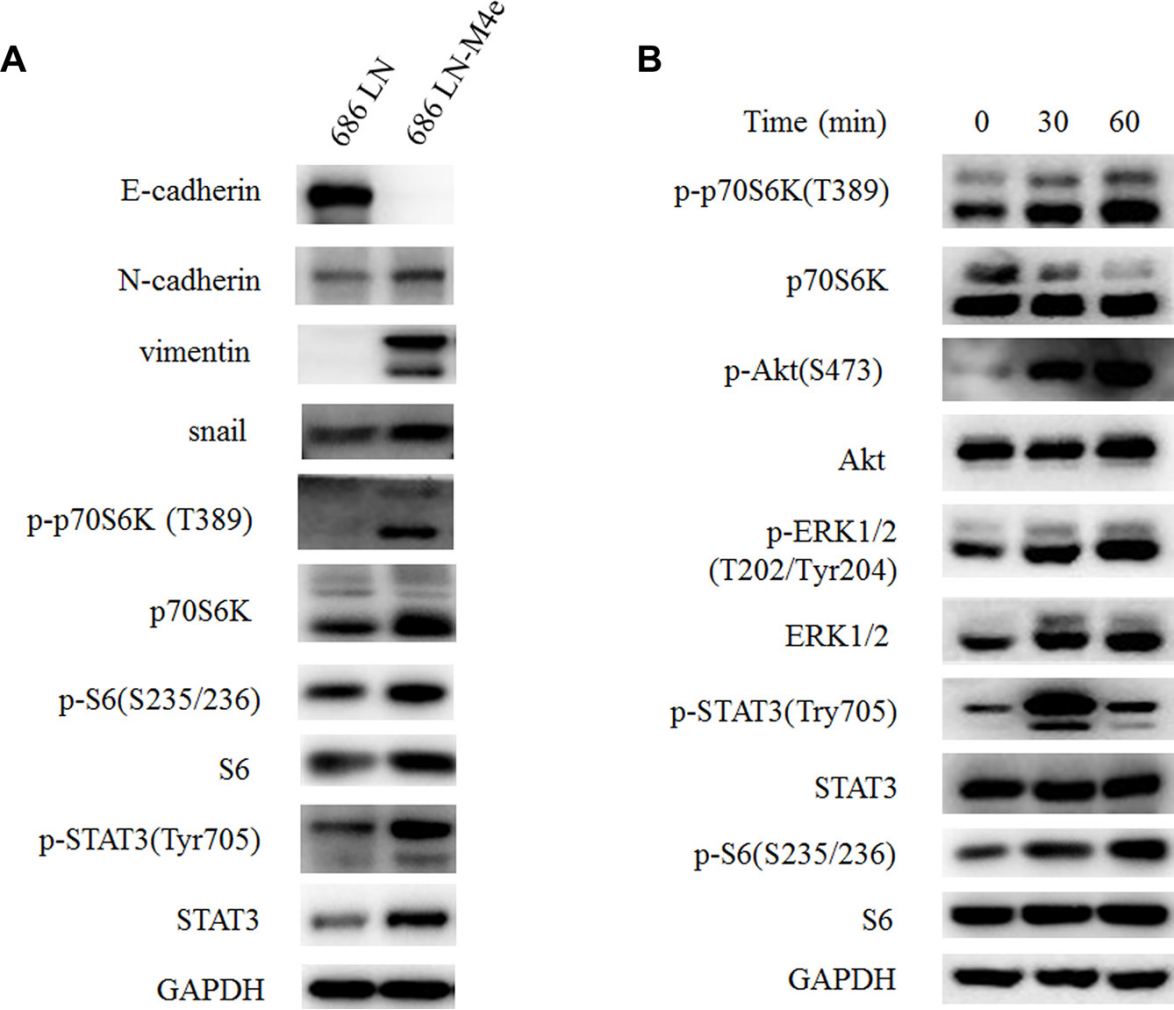
p70S6K is upregulated in 686LN-M4e cells compared to 686LN cells, and IL-6 activates p70S6K (**A**) 686LN cells and 686LN-M4e cells were seeded to 10 mm dishes for 24 hours. (**B**) 686LN cells were serum starved overnight, then treated with IL-6 50 ng/ml for different times, as indicated. Whole-cell protein lysates were prepared and subjected to western blotting.

We and other groups have previously reported that high-metastatic 686LN-M4e cells gained some EMT features compared to 686LN cells [23, 24]. In this study, western blot assay confirmed our previous findings that the protein levels of E-cadherin decreased, while N-cadherin, vimentin, and snail increased in 686LN-M4e cells, as compared with 686LN cells (Figure 2A). Concomitantly, we detected increased p-p70S6K, p-S6, and total p70S6K protein levels in 686LN-M4e cells, suggesting that p70S6K was activated and upregulated in parallel with EMT and the metastasis of HNSCCs (Figure 2A). We then investigated the effect of IL-6 on p70S6K. 686LN cells were treated with IL-6 for 30 and 60 minutes; p-p70S6K and p-S6 increased significantly, suggesting activation of p70S6K. We also examined other well-known signaling pathways that mediated IL-6/IL-6R signaling, such as PI3K/Akt, MAPK/ERK, and JAK/STAT3. Consistent with the findings of Yadav et al., p-Akt, p-ERK, and p-STAT3 were all increased with IL-6 treatment (Figure 2B) [8]. These results suggest that activation of p70S6K may mediate IL-6-induced EMT and the metastasis of HNSCCs.

### Overexpression of p70S6K promotes EMT and the migration of HNSCC cells

**Figure 3:**
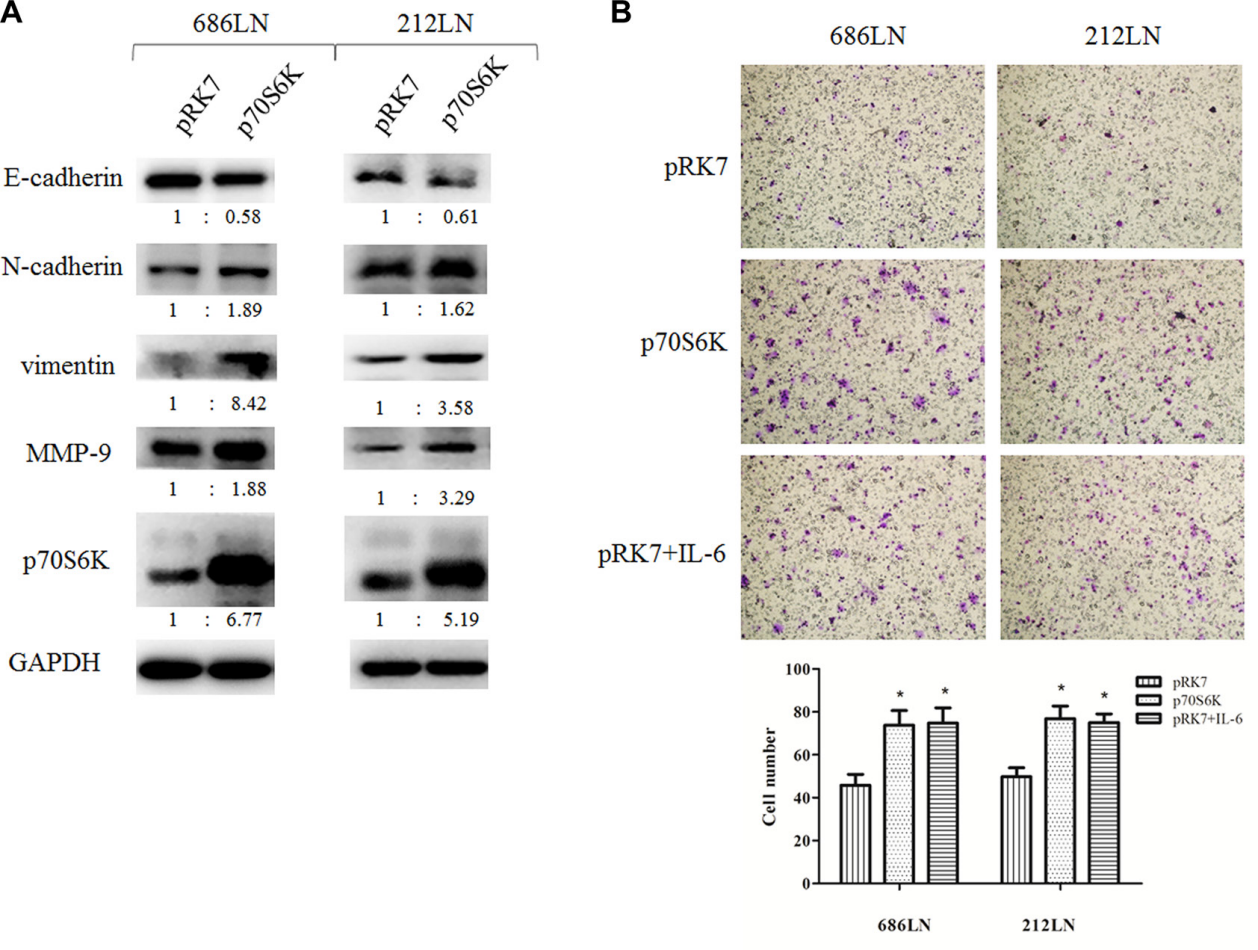
p70S6K induces EMT and migration (**A**) 686LN and 212LN cells were transfected with vector (pRK7) or p70S6K wild type constructs (p70S6K), as indicated, for 48 hours. Then whole-cell protein lysates were prepared and subjected to western blotting. The fold change of each treatment vs. the control was calculated after quantification and presented under each blot. (**B**) 686LN and 212LN cells were transfected with vector, p70S6K wild type constructs, or treated with 50 ng/ml IL-6, as indicated, for 24 hours. Then cells were subjected to a transwell assay. Magnification: 100×. Columns, means of cell number in five selected fields; bars, SD. *p < 0.05.

p70S6K has been reported to induce EMT in ovarian cancer cells, but its role in HNSCC is unclear [22]. Thus, we first evaluated the effect of p70S6K overexpression on EMT and the migration of HNSCC cells. 686LN and 212LN cells were transfected with constructs that encode wild type p70S6K (pRK7-p70S6K) or the control vector pRK7. After the 48 h transfection, the p70S6K protein levels increased 6.77 and 5.19 folds in 686LN and 212LN, respectively, confirming successful overexpression in both cell lines (Figure 3A). We found that E-cadherin decreased, while N-cadherin and vimentin increased, based on quantification of the immunoblot bands, suggesting that p70S6K induced EMT (Figure 3A). We also observed increased expression of MMP-9 in this experiment, suggesting that it may mediate p70S6K's effects (Figure 3A). Furthermore, transwell assay showed that cell migration increased significantly with wild type p70S6K constructs transfection or IL-6 treatment (Figure 3B). These results suggest that exogenous overexpression of p70S6K promotes EMT and the migration of HNSCC cells.

### Knockdown of p70S6K expression inhibited the IL-6-induced EMT and the migration of HNSCC cells

**Figure 4:**
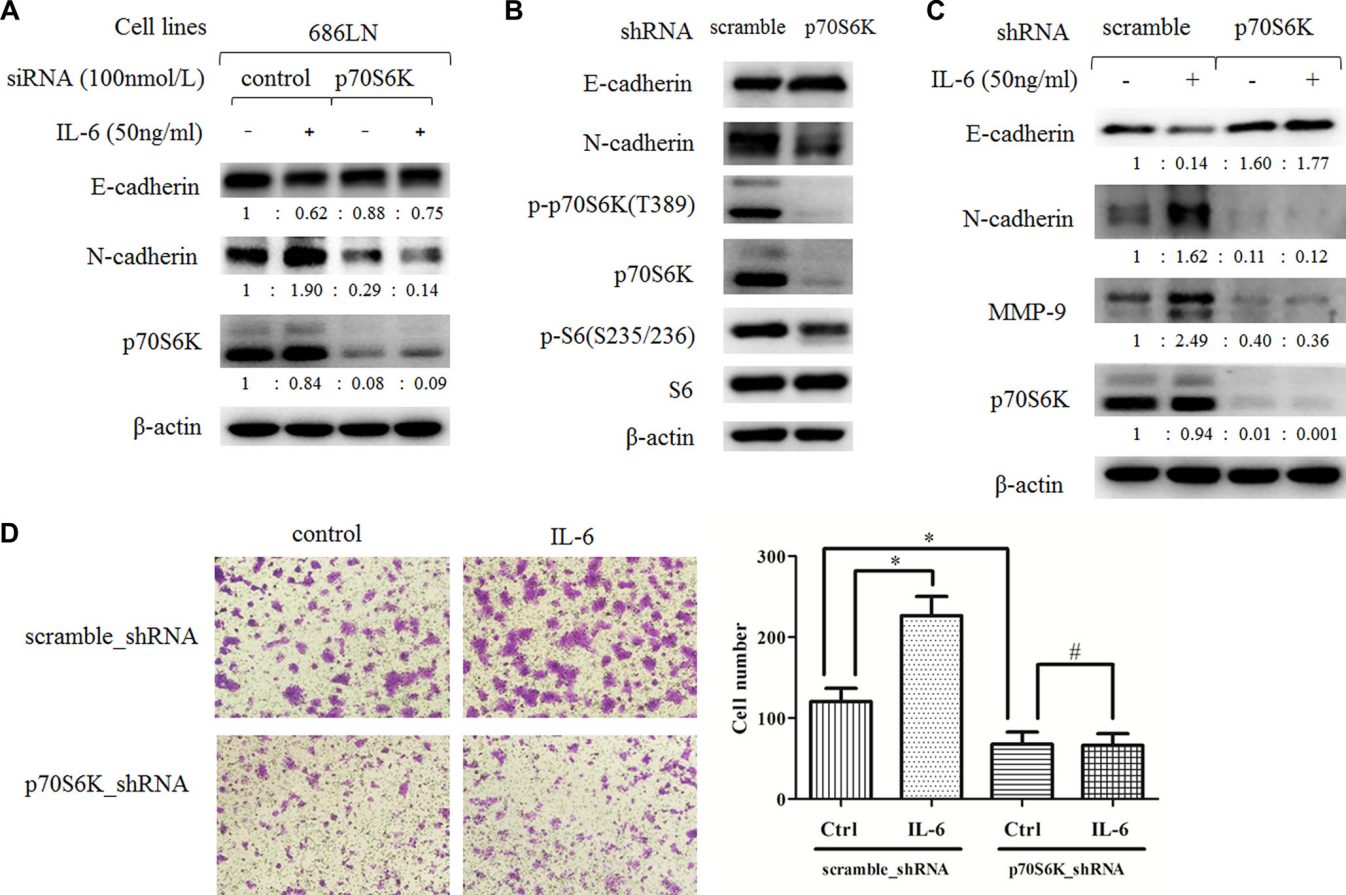
Downregulation of p70S6K expression inhibits IL-6-induced EMT and migration (**A**) 686LN cells were transfected with p70S6K siRNAs or control siRNAs for 24 hours, then they were serum starved overnight and treated with 50 ng/ml IL-6 for another 48 hours. (**B** and **C**) stable 686LN cell lines with p70S6K knockdown (p70S6K_shRNA) or the control cell lines (scramble_shRNA) were harvested directly (B) or harvested after being serum starved overnight and subsequently treated with IL-6 for 48 hours (C). Whole-cell protein lysates were prepared and subjected to western blotting. The fold change of each treatment vs. the control was calculated after quantification and presented under each blot. (**D**) transwell assay of stable cell lines, previously mentioned, with or without 50 ng/ml IL-6 for 24 hours. Magnification: 100×. Columns, means of cell number in five selected fields; bars, SD. *p < 0.05, ^#^*p* > 0.05.

We then examined whether p70S6K mediated IL-6-induced EMT and cell migration. We used p70S6K siRNAs (a pool of four target sequences) to knockdown p70S6K expression, and then we tested the effects of IL-6 on EMT. As shown in Figure 4A, p70S6K siRNAs significantly decreased p70S6K protein levels (about 90%), suggesting a successful silencing. IL-6 decreased E-cadherin and increased N-cadherin levels in control siRNAs transfected cells, but this effect was attenuated in p70S6K siRNAs transfected cells, suggesting that knockdown of p70S6K expression counteracted the IL-6's effect on EMT. We further confirmed these findings in p70S6K stable silencing cell lines, established by infection of lentivirus carrying p70S6K shRNA or scramble shRNA and subsequent puromycin selection for 14 days. As shown in Figure 4B, p-p70S6K, p70SK6, and p-S6 levels decreased significantly in 686LN-p70S6K_shRNA cells compared to 686LN-scramble_shRNAs cells, confirming successful knockdown of p70S6K. In parallel, E-cadherin increased and N-cadherin decreased in 686LN-p70S6K_shRNA cells, suggesting the p70S6K knockdown cell lines underwent mesenchymal-epithelial transition (MET), which is an opposing process to EMT (Figure 4B). Similarly, IL-6 induced a decrease of E-cadherin and an increase of N-cadherin and MMP-9 in 686LN-scramble_shRNAs cells, but this effect was attenuated in 686LN-p70S6K_shRNA cells (Figure 4C). Finally, transwell assay showed that IL-6 promoted cell migration in 686LN-scramble_shRNA cells. 686LN-p70S6K_shRNA cells showed decreased migratory ability compared to 686LN-scramble_shRNA cells, and the effect of IL-6 was significantly attenuated in p70S6K stable silencing cells (Figure 4D). These results suggest that knockdown of p70S6K expression counteracts IL-6-induced EMT and the migration of HNSCCs, as well as IL-6-induced MMP-9 expression.

### p70S6K located downstream of the PI3K/Akt/mTOR and MAPK/ERK signaling pathways to mediate IL-6's effect

**Figure 5:**
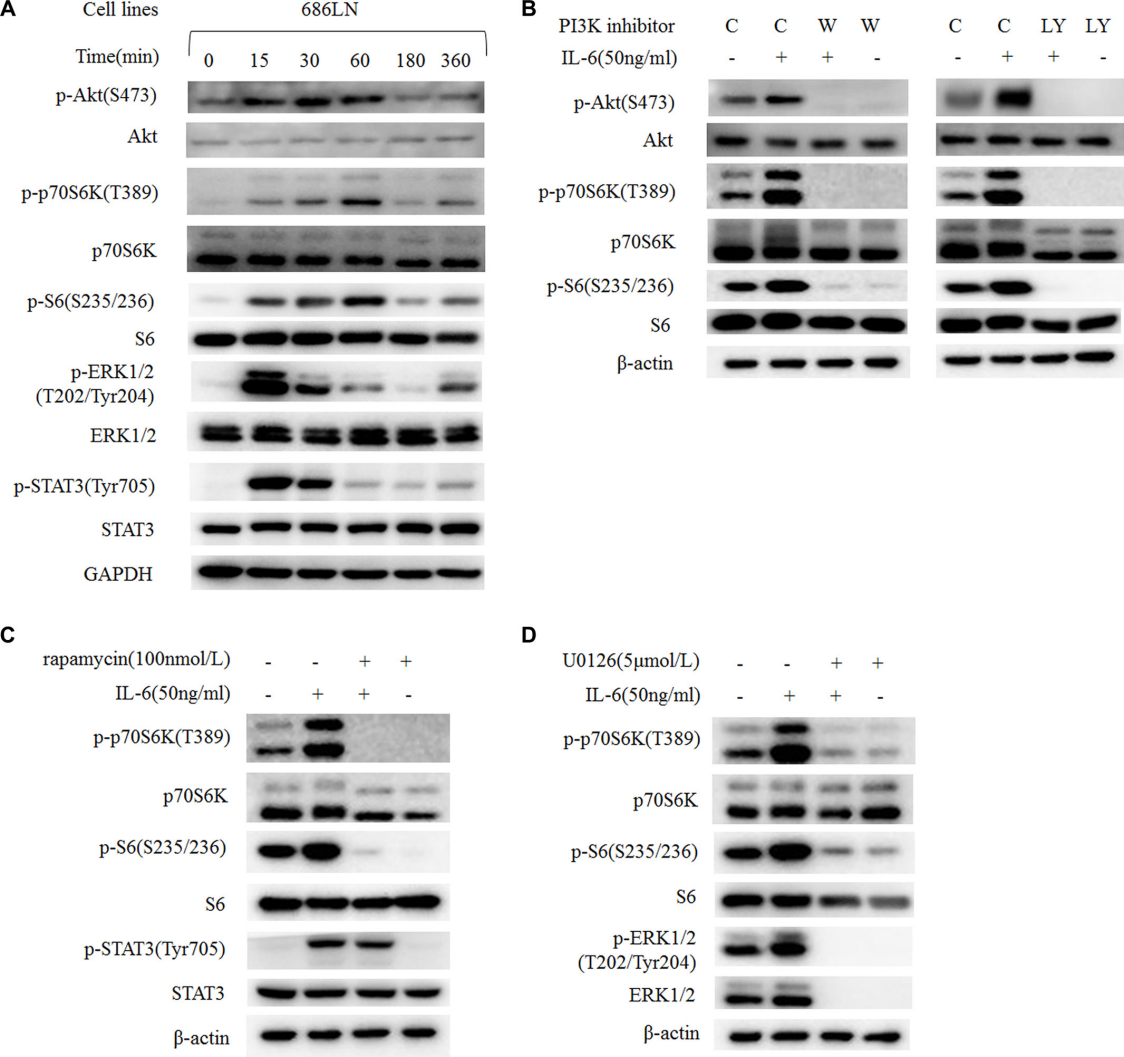
IL-6-induced activation of p70S6K locates downstream of the PI3K/Akt/mTOR and MAPK signaling pathways (**A**) 686LN cells were serum starved overnight and treated with 50 ng/ml IL-6 for different times, as indicated. (**B**, **C**, and **D**) 686LN cells were serum starved overnight and pretreated with different inhibitors: wortmannin 250 nmol/L (B), LY294002 20 μmol/L (B), rapamycin 100 nmol/L (C), or U0126 5 μmol/L (D) for 30 minutes, then co-treated with or without IL-6 50 ng/ml for another hour. Whole-cell protein lysates were prepared and subjected to western blotting. *C, control; W, wortmannin; LY, LY294002*.

The PI3K/Akt/mTOR and MAPK/ERK signaling pathways are acknowledged as the main signaling pathways that mediate IL-6's stimulating effect in many cancer types [8, 10, 25]. Even though p70S6K locates downstream of the PI3K/Akt/mTOR signaling pathway, there is some crosstalk between these pathways. Therefore, we explored which pathway mediated the phosphorylation and activation of p70S6K. First, a time-course study (0–360 minutes) of IL-6 treatment in 686LN cells showed that the peak of increased p-ERK and p-STAT3 was 15 minutes, p-Akt was 30–60 minutes, and p-p70S6K and p-S6 was 60 minutes, suggesting that p70S6K activation happened later than other events (Figure 5A). Similar results were detected in 212LN cell lines (Supplementary Figure S1). Then we found that the PI3K inhibitors LY294002 and wortmannin decreased basal p-p70S6K and p-S6 levels, as well as IL-6-induced expression levels, suggesting that p70S6K located downstream of the PI3K/Akt signaling pathway (Figure 5B). Similar results were found when using the mTOR inhibitor rapamycin (Figure 5C) and the MEK1/2 inhibitor U0126 (Figure 5D). These results suggest that p70S6K activation locates downstream of the PI3K/Akt/mTOR and MAPK/ERK signaling pathways after IL-6 stimulation.

### IL-6-induced activation of p70S6K was also mediated by STAT3

**Figure 6:**
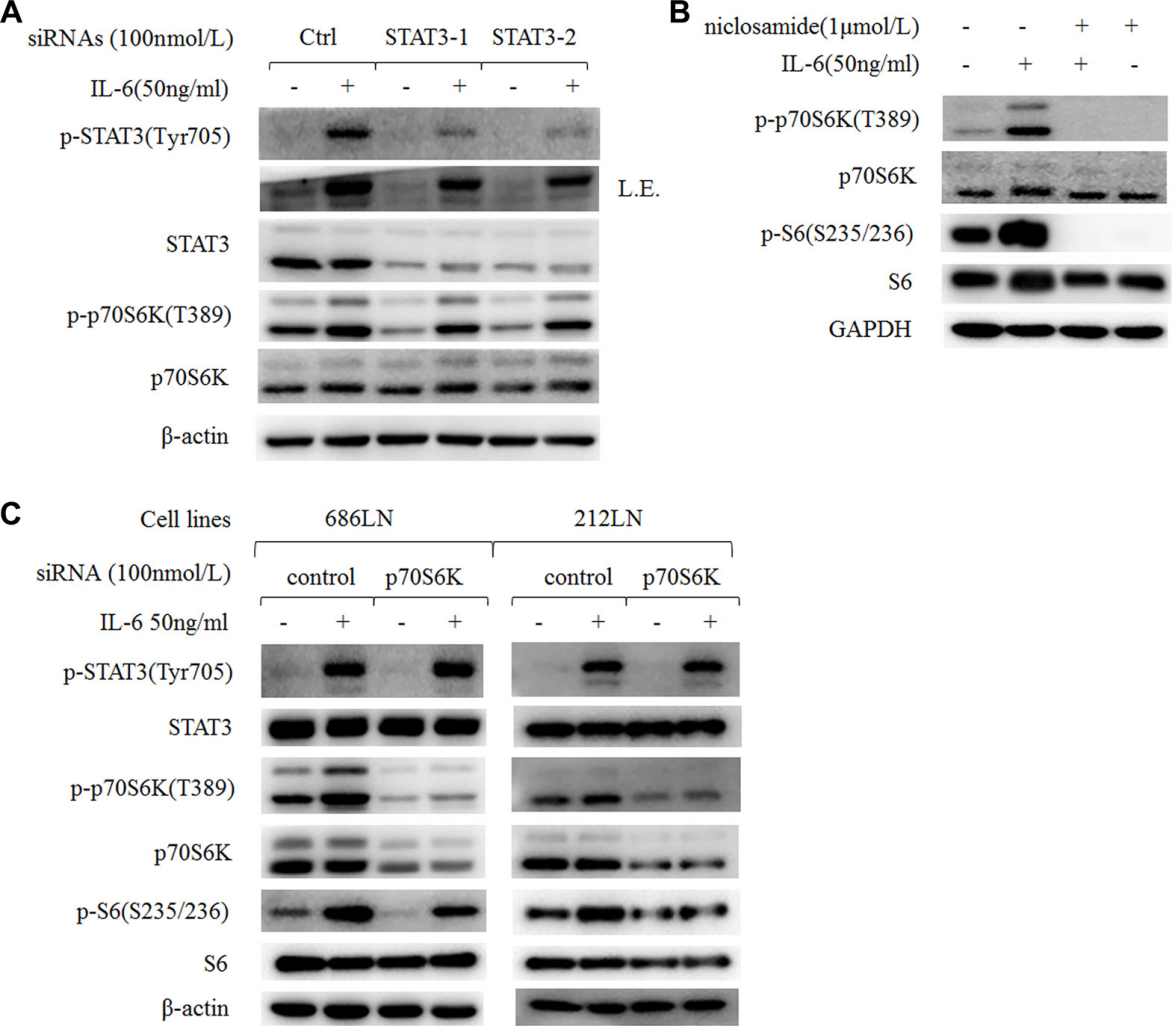
IL-6-induced activation of p70S6K locates downstream of STAT3 (**A**) 686LN cells were transfected with two different sequences of STAT3 siRNAs (STAT-1 and STAT-2) or control siRNAs for 24 hours, then they were serum starved overnight and treated with or without 50 ng/ml IL-6 for another hour. (**B**) 686LN cells were serum starved overnight and pretreated with niclosamide 1 μmol/L for 30 minutes, then co-treated with or without IL-6 50 ng/ml for another hour. (**C**) 686LN and 212LN cells were transfected with p70S6K pool siRNAs (four sequences) or control siRNAs for 24 hours, then were serum starved overnight and treated with or without IL-6 50 ng/ml for another hour. Whole-cell protein lysates were prepared and subjected to western blot analysis. *L.E., long exposure*.

STAT3 is one of the key molecules in malignancy and the metastasis of HNSCCs [4, 26]. We also found increased p-STAT3 in 686LN-M4e cells compared to 686LN cells (Figure 2A). And IL-6 induced a significant increase of p-STAT3 in HNSCC cells earlier than the p-p70S6K increase (Figures 2B and 5A). It has been reported that STAT3 locates downstream of mTOR, but its relationship with p70S6K is unknown [27, 28]. Using two target sequences of siRNAs to knockdown STAT3 expression, we found that p-p70S6K decreased as STAT3 silenced; however, for IL-6-induced p70S6K, it was only partially attenuated (Figure 6A). Since there were less than 30% STAT3 protein left after silencing, and IL-6 increased the phosphorylation of the left STAT3 to some degree, we suspect that these STAT3 proteins might function to activate p-p70S6K. Therefore, an inhibitor of STAT3, niclosamide, was used to block the activity of STAT3 without affecting its global protein levels. Niclosamide decreased p-p70S6K levels as well as inhibited IL-6-induced p-p70S6K, suggesting that p70S6K located downstream of STAT3 to mediate IL-6's signal (Figure 6B).

In addition, we found that rapamycin decreased basal p-STAT3 levels and IL-6-induced p-STAT3; this was consistent with other reports that mTOR activated STAT3 (Figure 5C). However, using siRNAs to knockdown p70S6K expression did not affect IL-6-induced p-STAT3 (Figure 6C). These findings suggest that there is crosstalk among STAT3, mTOR, and p70S6K, but p70S6K locates downstream of STAT3 and mTOR in mediating IL-6's signaling.

## DISCUSSION

In this study, we proved for the first time that p70S6K promoted EMT and the metastasis of HNSCCs based on the following findings: 1) paired high-metastatic HNSCC cells underwent EMT in parallel with p70S6K upregulation and activation; 2) overexpression of p70S6K induced EMT and promoted the migration of HNSCC cells; 3) IL-6 activated p70S6K, and the knockdown of p70S6K attenuated IL-6-induced EMT and cell migration. Many studies have reported the role of p70S6K in metastasis within the mTOR signaling pathway, including mTORC1 and mTORC2 [17]. For example, activation of the mTOR signaling pathway promotes, while blocking this pathway inhibits, EMT, cell migration, invasion, and cancer metastasis [19, 29, 30]. Among numerous evaluations of the expression profiles of the PI3K/Akt/mTOR signaling components in human tissue samples, p70S6K upregulation and activation have been proven to be correlated with tumor malignancy, metastasis, and poor patient survival [17]. However, the intensive studies focusing on the role of p70S6K in EMT and in metastasis of specific cancer types are very limited.

p70S6K is reported to regulate cytoskeletal organization and cell motility induced by Rho GTPase family members, such as Rho A, Rac1, and cdc42 [31, 32]. In ovarian cancer, it promotes EMT, invasion, and metastasis through inducing snail expression [22]. It induces MMP-9 expression and is associated with hepatocyte growth factor-mediated invasion of ovarian cancer cells [33]. In this study, we observed that overexpression of p70S6K increased MMP-9 expression, while knockdown of p70S6K decreased MMP-9 expression, suggesting that p70S6K-mediated upregulation of MMP-9 was involved in the IL-6-induced metastasis of HNSCCs. Even though p70S6K is suggested to be involved in translation initiation, elongation, pre-mRNA splicing, and protein folding by targeting various signals, it is generally considered as a global regulatory factor [17]. For example, it phosphorylates and activates the 40S ribosomal subunit protein S6 to promote ribosome translation; therefore, p-S6 was often used as a marker for p70S6K activity. Other molecules that mediate its function for biosynthesis include eIF3, eIF4B, eEF2K, PDCD4, TRAF4, SKAR1, CCTβ, CBP80, and MDM2 [17]. Currently, more and more interests have arisen for identifying new downstream targets of p70S6K that regulate transcription and translation of specific oncogene or tumor suppressors, as well as new crosstalk with other kinases and feedback loops.

**Figure 7:**
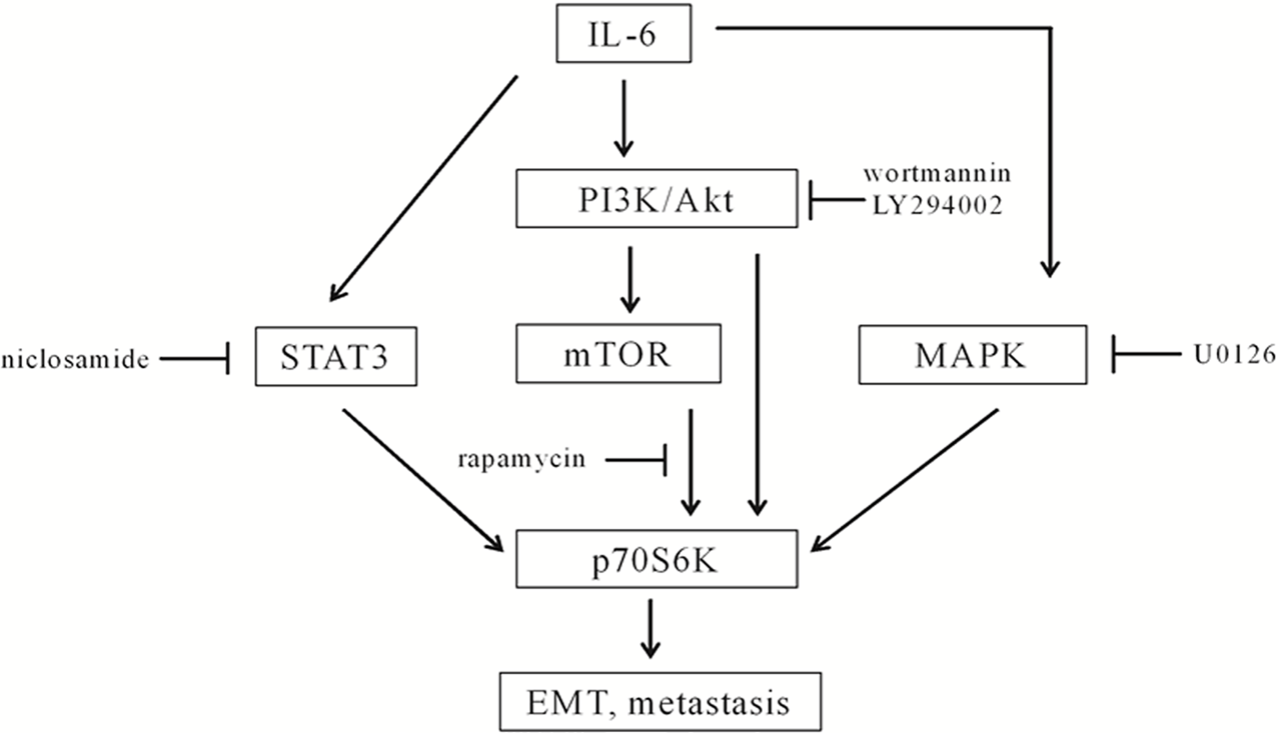
A diagram of IL-6-induced activation of p70S6K in the metastasis of HNSCCs IL-6 activated the PI3K/Akt/mTOR, MAPK, and STAT3 signaling pathways. p70S6K located downstream of the PI3K/Akt/mTOR, MAPK, and STAT3 signaling pathways, because wortmannin, LY294002, rapamycin, U0126, and niclosamide inhibited IL-6-induced activation of p70S6K. p70S6K was a node for multiple signaling pathways to mediate IL-6-induced EMT and the metastasis of HNSCCs.

In recent years, the relationship between cancer and immune response and inflammation has attracted more and more attention. IL-6 is a cytokine that has been extensively studied in cancer research. Upregulated IL-6/STAT3 signaling pathways and elevated IL-6 serum levels are associated with the malignance, metastasis, and drug sensitivity of HNSCCs [12–14]. In this study, we identified p70S6K activation by IL-6 located downstream of the PI3K/Akt/mTOR, MAPK/ERK, and JAK/STAT3 signaling pathways, which were the most important survival pathways that promote most cancers. Currently, more and more studies are being performed with high-throughput technologies and high-powered computational assays, such as microarray, DNA methylation, protein array, miRNA profiling, and bioinformatic analysis. The PI3K/Akt/mTOR, IL-6/IL-6R/JAK/STAT3, EGFR/MAPK/AP1 signaling pathways are suggested to be the main factors and pathways in HNSCCs [4]. Yadav et al. report that IL-6 promotes metastasis of HNSCC through induction of EMT via the JAK-STAT3-SNAIL signaling pathway [8]. We observed that IL-6-activated p70S6K was repressed by PI3K/Akt/mTOR inhibitors, MAPK inhibitors, and STAT3 inhibitors. Furthermore, silencing p70S6K eliminated IL-6-induced EMT and cell migration. We suspect that p70S6K was a node or output for multiple signaling pathways (Figure 7). Therefore, targeting p70S6K for cancer therapy may not only shrink the original tumor size but also decrease the potential for metastasis and recurrence.

In summary, we found that p70S6K plays an important role in promoting EMT and the metastasis of HNSCCs. We also showed that under IL-6-stimulating conditions, activation of p70S6K locates downstream of the PI3K/Akt/mTOR, MAPK/ERK, and JAK/STAT3 signaling pathways. These findings suggest that targeting p70S6K in HNSCC therapy may not only inhibit tumor growth but also have the potential to block metastasis.

## MATERIALS AND METHODS

### Reagents

Recombinant Human Interleukin-6 (rHuIL-6; PR1073) was purchased from Bioworld Technology, Inc. (Louis Park, MN). Wortmannin (S1952), LY294002 (S1737), and U0126 (S1901) were purchased from the Beyotime Institute of Biotechnology (Jiangsu, China). Rapamycin (R-5000) was purchased from LC Laboratories. Niclosamide (N-3510-50G) and puromycin dihydrochloride (P8833) were purchased from Sigma Chemical, Co. (St. Louis, MO). Lipofectamine 2000 transfection reagent (11668–019) and Trizol reagent (15596018) were purchased from Life Technologies Co. Invitrogen (Carlsbad, CA, USA). E-cadherin (R868) pAb (BS1098), N-cadherin (W745) pAb (BS2224), β-actin mAb (BS6007M), GAPDH pAb (AP0063), and ERK1/2 (D196) pAb (BS3627) were purchased from Bioworld Technology, Inc. (Louis Park, MN). Rabbit Vimentin Polyclonal Antibody (10366-1-AP) and Rabbit SNAI1 Polyclonal Antibody (13099-1-AP) were purchased from Proteintech Group, Inc. (Chicago, IL). p-p70S6K(Thr389) (9205), p-S6(Ser235/236) (4858), p-STAT3(Tyr705), p-ERK1/2(Thr202/Tyr204) (9101), p-Akt(Ser473) (9271), p70S6K (9202), S6 (2217), STAT3 (12640), and Akt (9272) antibodies were purchased from Cell Signaling Technology, Inc. (Beverly, MA).

### Cell lines and cell culture

686LN, 686LN-M4e, and 212LN were kind gifts from Dr. Georgia Chen of Emory University. Paired low- and high-metastatic HNSCC cells, 686LN and 686LN-M4e, have been previously described [23]. Briefly, metastatic 686LN cells were collected from cervical lymph nodes of nude mice with 686LN cells injected into the mylohyoid muscle near the FOM (floor-of-mouth). After four rounds of *in vivo* selection, the high-metastatic 686LN-M4e cell line was established, and it showed 100% lymph node metastasis and 90% lung metastasis in nude mice *in vivo*. 686LN-M4e also showed much higher Matrigel invasion capability than the parental cells [34, 35]. These cell lines were grown in a monolayer cultured in Dulbecco's modified Eagle's medium (DMEM)/F12 medium supplemented with 10% fetal bovine serum at 37°C in a humidified atmosphere consisting of 5% CO2 and 95% air.

### Western blot analysis

Whole-cell protein lysis buffer, containing 1% Triton, 40 mmol/l HEPES, 120 mmol/l NaCl, 1 mmol/l EDTA, 10 mmol/l pyrophosphate, 10 mmol/l glycerophosphate, 50 mmol/l NaF, and 0.5 mmol/l N_a_3VO4, was prepared and stored at 4°C. Protease inhibitors were added just before use. The procedures for western blotting were described previously. Western Lightening^®^ Plus-ECL, Enhanced Chemiluminescence Substrate (NEL103001EA) from PerkinElmer, Inc. (Waltham, MA, USA) was used for exposure [36, 37]. The chemiluminescent signal was collected and analyzed by Image J plus 6.0. The Index of Density (IOD) of each band = density × area. The value of IOD ratio (IOD ratio = IOD of target gene/IOD of house-keeping gene) was calculated. The fold change (Fold change = IOD ratio of treatment/IOD ratio of control) was presented under each blot.

### RNA isolation and quantitative reverse transcription polymerase chain reaction (qRT-PCR)

Total RNAs from cells were extracted using Trizol reagent, and reverse transcription was conducted according to the standard procedure using Thermo Scientific RevertAid Reverse Transcriptase (EP0442; Rockford, IL). PCR reactions were conducted according to the manufacturer's instructions using FastStart Universal SYBR Green Master (Rox; 14928100) from Roche Diagnostics (Indianapolis, IN). Forward (F) and reverse (R) primers were previously described: IL-6, F: 5′- AAC CTG AAC CTT CCA AAG ATG G −3′ and R: 5′- TCT GGC TTG TTC CTC ACT ACT −3′; GAPDH, F: 5′-ATG GGG AAG GTG AAG GTC G-3′, and R: 5′-GGG GTC ATT GAT GGC AAC AAC AAT A-3′. They were synthesized by Invitrogen Life Technologies [16, 38]. All real-time amplifications were measured in triplicate and performed with the ABI Prism 7300 sequence detection system (Applied Biosystems). The fold-change of mRNAs was calculated using the 2^−ΔΔCT^ method.

### Transwell migration assay

Cell migration was evaluated using a 24-well Millicell Hanging Cell Culture Inserts (8.0-μm pore size; PIEP12R48) from Millipore Corporation (Billerica, MA). Briefly, 2–5 × 10^4^ cells were seeded into the inside of the inserts and serum starved overnight. Then the outside of the inserts were replaced with condition medium, which was harvested from cultured NIH3T3 cells, containing IL-6 (50 ng/ml) or its vehicle control. After culturing for 24 hours, the non-migratory cells in the inserts were removed with a cotton swab, and the migratory cells on the outside of the inserts were fixed with methanol and stained with 0.1% crystal violet. The migrated cells on the lower surface of the membrane were photographed, and the cell numbers were counted under a microscope. Five fields from each sample were selected under a microscope with 100× magnification. The average numbers of migrated cells under each field were counted and presented as mean ± SD. Representative fields of migrated cells with 100× magnification were presented.

### Gene silencing by RNA interfering (RNAi)

Control (non-target) small interfering RNA (siRNA) was purchased from Shanghai GenePharma. p70S6K siRNA pools that target 5′-CCA AGG UCA UGU GAA ACU A-3′; 5′-CAU GGA ACA UUG UGA GAA A-3′; 5′-GAC GGG GUC CUC AAA UGU A-3′; 5′-GCA GGA GUG UUU GAC AUA G-3′ [22] and STAT3 siRNAs that target 5′-CAU CUG CCU AGA UCG GCU ATT -3′; 5′-CCA CUU UGG UGU UUC AUA ATT-3′ [39, 40] were previously described, and they were synthesized by Shanghai GenePharma. Cells were seeded to a 6-well plate at 5 × 10^5^ cells/well and transfected with 100 nmol/L control, p70S6K, or STAT3 siRNAs using Lipofectamine 2000 following its instruction, and they were then subjected to subsequent assays after 48 hours.

### Establishing of stable cell lines

Two target sequences of p70S6K siRNAs were selected for constructing the lentivirus shRNA plasmids. Control lentivirus (carrying scramble shRNAs) and p70S6K lentiviruses (carrying p70S6K shRNAs) were established by Shanghai GeneChem Co., Ltd. Two p70S6K shRNA lentiviruses were pooled together and infected the 686LN cells. Cell lines were then selected by culturing with 5 μg/ml puromycin dihydrochloride beginning 48 hours after infection, and they were cultured for two weeks. They were then further cultured for another two weeks without puromycin. The expression of p70S6K was confirmed by western blot analysis.

### Gene overexpression by plasmids

The pRK7-p70S6K plasmid (encoding wild type form) and its control plasmid pRK7 were described previously and kindly provided by Dr. John Blenis (Harvard Medical School) [41]. Transfection was conducted using Lipofectamine 2000, as we previously described [36].

### Statistical analysis

All data were presented as the mean ± SD and was representative of the three independent experiments. The statistical significance of the different groups was analyzed using the two-sided unpaired Student's *t*-tests. GraphPad software was used for data analysis. The results were considered to be statistically significant at *p* < 0.05.

## SUPPLEMENTARY MATERIALS FIGURES



## References

[1] Gaykalova DA, Manola JB, Ozawa H, Zizkova V, Morton K, Bishop JA, Sharma R, Zhang C, Michailidi C, Considine M, Tan M, Fertig EJ, Hennessey PT (2015). NF-kappaB and stat3 transcription factor signatures differentiate HPV-positive and HPV-negative head and neck squamous cell carcinoma. Int J Cancer.

[2] Leemans CR, Braakhuis BJ, Brakenhoff RH (2011). The molecular biology of head and neck cancer. Nat Rev Cancer.

[3] Kalavrezos N, Bhandari R (2010). Current trends and future perspectives in the surgical management of oral cancer. Oral Oncol.

[4] Yan B, Broek RV, Saleh AD, Mehta A, Van Waes C, Chen Z (2013). Signaling Networks of Activated Oncogenic and Altered Tumor Suppressor Genes in Head and Neck Cancer. J Carcinog Mutagen.

[5] Thiery JP, Acloque H, Huang RY, Nieto MA (2009). Epithelial-mesenchymal transitions in development and disease. Cell.

[6] Thiery JP (2002). Epithelial-mesenchymal transitions in tumour progression. Nat Rev Cancer.

[7] Brabletz T (2012). To differentiate or not—routes towards metastasis. Nat Rev Cancer.

[8] Yadav A, Kumar B, Datta J, Teknos TN, Kumar P (2011). IL-6 promotes head and neck tumor metastasis by inducing epithelial-mesenchymal transition via the JAK-STAT3-SNAIL signaling pathway. Mol Cancer Res.

[9] Lauta VM (2001). Interleukin-6 and the network of several cytokines in multiple myeloma: an overview of clinical and experimental data. Cytokine.

[10] Wang Y, Niu XL, Qu Y, Wu J, Zhu YQ, Sun WJ, Li LZ (2010). Autocrine production of interleukin-6 confers cisplatin and paclitaxel resistance in ovarian cancer cells. Cancer Lett.

[11] Lee TL, Yeh J, Van Waes C, Chen Z (2006). Epigenetic modification of SOCS-1 differentially regulates STAT3 activation in response to interleukin-6 receptor and epidermal growth factor receptor signaling through JAK and/or MEK in head and neck squamous cell carcinomas. Mol Cancer Ther.

[12] Riedel F, Zaiss I, Herzog D, Gotte K, Naim R, Hormann K (2005). Serum levels of interleukin-6 in patients with primary head and neck squamous cell carcinoma. Anticancer Res.

[13] Duffy SA, Taylor JM, Terrell JE, Islam M, Li Y, Fowler KE, Wolf GT, Teknos TN (2008). Interleukin-6 predicts recurrence and survival among head and neck cancer patients. Cancer.

[14] Hong DS, Angelo LS, Kurzrock R (2007). Interleukin-6 and its receptor in cancer: implications for translational therapeutics. Cancer.

[15] Pu YS, Hour TC, Chuang SE, Cheng AL, Lai MK, Kuo ML (2004). Interleukin-6 is responsible for drug resistance and anti-apoptotic effects in prostatic cancer cells. Prostate.

[16] Yao Z, Fenoglio S, Gao DC, Camiolo M, Stiles B, Lindsted T, Schlederer M, Johns C, Altorki N, Mittal V, Kenner L, Sordella R (2010). TGF-beta IL-6 axis mediates selective and adaptive mechanisms of resistance to molecular targeted therapy in lung cancer. Proc Natl Acad Sci U S A.

[17] Magnuson B, Ekim B, Fingar DC (2012). Regulation and function of ribosomal protein S6 kinase (S6K) within mTOR signalling networks. Biochem J.

[18] Abe Y, Yoon SO, Kubota K, Mendoza MC, Gygi SP, Blenis J (2009). p90 ribosomal S6 kinase and p70 ribosomal S6 kinase link phosphorylation of the eukaryotic chaperonin containing TCP-1 to growth factor, insulin, and nutrient signaling. J Biol Chem.

[19] Zhou H, Huang S (2011). Role of mTOR signaling in tumor cell motility, invasion and metastasis. Curr Protein Pept Sci.

[20] Bancroft CC, Chen Z, Yeh J, Sunwoo JB, Yeh NT, Jackson S, Jackson C, Van Waes C (2002). Effects of pharmacologic antagonists of epidermal growth factor receptor, PI3K and MEK signal kinases on NF-kappaB and AP-1 activation and IL-8 and VEGF expression in human head and neck squamous cell carcinoma lines. Int J Cancer.

[21] Herzog A, Bian Y, Vander Broek R, Hall B, Coupar J, Cheng H, Sowers AL, Cook JD, Mitchell JB, Chen Z, Kulkarni AB, Van Waes C (2013). PI3K/mTOR inhibitor PF-04691502 antitumor activity is enhanced with induction of wild-type TP53 in human xenograft and murine knockout models of head and neck cancer. Clin Cancer Res.

[22] Pon YL, Zhou HY, Cheung AN, Ngan HY, Wong AS (2008). p70 S6 kinase promotes epithelial to mesenchymal transition through snail induction in ovarian cancer cells. Cancer Res.

[23] Zhang X, Su L, Pirani AA, Wu H, Zhang H, Shin DM, Gernert KM, Chen ZG (2006). Understanding metastatic SCCHN cells from unique genotypes to phenotypes with the aid of an animal model and DNA microarray analysis. Clin Exp Metastasis.

[24] Luo X, Fan S, Huang W, Zhai S, Ma Z, Li P, Sun SY, Wang X (2012). Downregulation of IRS-1 promotes metastasis of head and neck squamous cell carcinoma. Oncol Rep.

[25] Rosich L, Montraveta A, Xargay-Torrent S, Lopez-Guerra M, Roldan J, Aymerich M, Salaverria I, Bea S, Campo E, Perez-Galan P, Roue G, Colomer D (2014). Dual PI3K/mTOR inhibition is required to effectively impair microenvironment survival signals in mantle cell lymphoma. Oncotarget.

[26] Lee TL, Yeh J, Friedman J, Yan B, Yang X, Yeh NT, Van Waes C, Chen Z (2008). A signal network involving coactivated NF-kappaB and STAT3 and altered p53 modulates BAX/BCL-XL expression and promotes cell survival of head and neck squamous cell carcinomas. Int J Cancer.

[27] He SQ, Gao M, Fu YF, Zhang YN (2015). Glycyrrhizic acid inhibits leukemia cell growth and migration via blocking AKT/mTOR/STAT3 signaling. Int J Clin Exp Pathol.

[28] Sakamoto KM, Grant S, Saleiro D, Crispino JD, Hijiya N, Giles F, Platanias L, Eklund EA (2015). Targeting novel signaling pathways for resistant acute myeloid leukemia. Mol Genet Metab.

[29] Liu L, Li F, Cardelli JA, Martin KA, Blenis J, Huang S (2006). Rapamycin inhibits cell motility by suppression of mTOR-mediated S6K1 and 4E-BP1 pathways. Oncogene.

[30] Gulhati P, Bowen KA, Liu J, Stevens PD, Rychahou PG, Chen M, Lee EY, Weiss HL, O'Connor KL, Gao T, Evers BM (2011). mTORC1 and mTORC2 regulate EMT, motility, and metastasis of colorectal cancer via RhoA and Rac1 signaling pathways. Cancer Res.

[31] Aslan JE, Tormoen GW, Loren CP, Pang J, McCarty OJ (2011). S6K1 and mTOR regulate Rac1-driven platelet activation and aggregation. Blood.

[32] Berven LA, Crouch MF (2000). Cellular function of p70S6K: a role in regulating cell motility. Immunol Cell Biol.

[33] Zhou HY, Wong AS (2006). Activation of p70S6K induces expression of matrix metalloproteinase 9 associated with hepatocyte growth factor-mediated invasion in human ovarian cancer cells. Endocrinology.

[34] Zhang H, Su L, Muller S, Tighiouart M, Xu Z, Zhang X, Shin HJ, Hunt J, Sun SY, Shin DM, Chen ZG (2008). Restoration of caveolin-1 expression suppresses growth and metastasis of head and neck squamous cell carcinoma. Br J Cancer.

[35] Zhang X, Liu Y, Gilcrease MZ, Yuan XH, Clayman GL, Adler-Storthz K, Chen Z (2002). A lymph node metastatic mouse model reveals alterations of metastasis-related gene expression in metastatic human oral carcinoma sublines selected from a poorly metastatic parental cell line. Cancer.

[36] Ma Z, Zhu L, Luo X, Zhai S, Li P, Wang X (2012). Perifosine enhances mTORC1-targeted cancer therapy by activation of GSK3beta in NSCLC cells. Cancer Biol Ther.

[37] Huang W, Yang L, Liang S, Liu D, Chen X, Ma Z, Zhai S, Li P, Wang X (2013). AEG-1 is a target of perifosine and is over-expressed in gastric dysplasia and cancers. Dig Dis Sci.

[38] Dubrovska A, Kim S, Salamone RJ, Walker JR, Maira SM, Garcia-Echeverria C, Schultz PG, Reddy VA (2009). The role of PTEN/Akt/PI3K signaling in the maintenance and viability of prostate cancer stem-like cell populations. Proc Natl Acad Sci U S A.

[39] Konnikova L, Kotecki M, Kruger MM, Cochran BH (2003). Knockdown of STAT3 expression by RNAi induces apoptosis in astrocytoma cells. BMC Cancer.

[40] Konnikova L, Simeone MC, Kruger MM, Kotecki M, Cochran BH (2005). Signal transducer and activator of transcription 3 (STAT3) regulates human telomerase reverse transcriptase (hTERT) expression in human cancer and primary cells. Cancer Res.

[41] Schalm SS, Blenis J (2002). Identification of a conserved motif required for mTOR signaling. Curr Biol.

