# Supplementary material for: p70S6K promotes IL-6-induced epithelial-mesenchymal transition and metastasis of head and neck squamous cell carcinoma
Source: Oncotarget. 2016 May 11;7(24):36539–50. doi: 10.18632/oncotarget.9282 (PMC5095019; doi:10.18632/oncotarget.9282)
Supplement: Supplementary file 1 [file oncotarget-07-36539-s001.pdf]

p70S6K promotes IL-6-induced epithelial-mesenchymal transition and metastasis of head and neck squamous cell carcinoma

Supplementary Materials

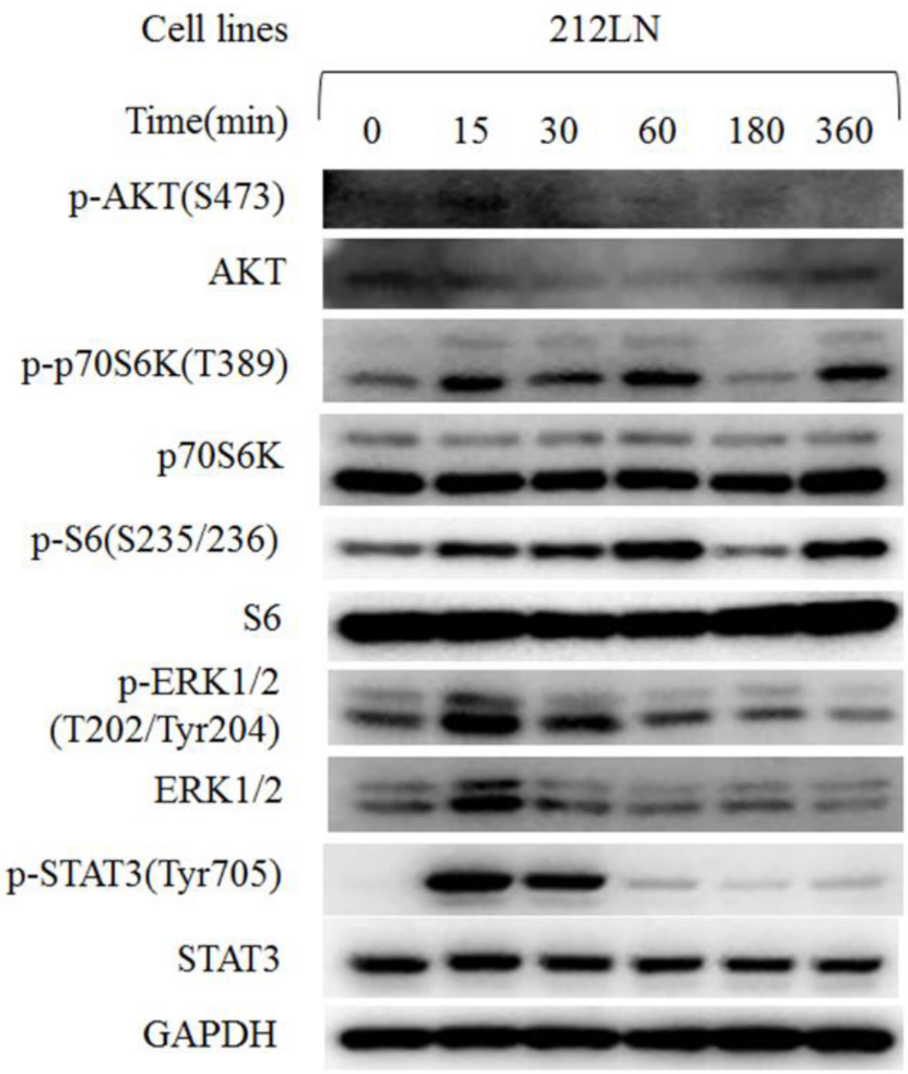

Supplementary Figure S1: IL-6 activities multiple signaling pathways. 212LN cells were treated with IL-6 for different times as indicated. Whole-cell protein lysates were prepared and subjected to western blotting.
